# Supplementary material for: Antimicrobial and Antibiofilm Activities of Some Antioxidant 3,4-Dihydroxyphenyl-Thiazole-Coumarin Hybrid Compounds: In Silico and In Vitro Evaluation
Source: Antibiotics (Basel). 2025 Sep 18;14(9):943. doi: 10.3390/antibiotics14090943 (PMC12466426; doi:10.3390/antibiotics14090943)
Supplement: Supplementary file 1 [file antibiotics-14-00943-s001.zip › antibiotics-3794765_supplementary.pdf]

antibiotics

Article

Supplementary Material: Antimicrobial and Antibiofilm Activities of Some Antioxidant 3,4-Dihydroxyphenyl-Thiazole-Coumarin Hybrid Compounds: In Silico and In Vitro Evaluation

Daniel Ungureanu, Gabriel Marc, Mihaela Niculina Duma, Radu Tamaian, Dan Cristian Vodnar, Brîndușa Tiperciuc, Cristina Moldovan, Ioana Ionuț, Anca Stana, Ovidiu Oniga

1. Figures

1.1. Molecular Dynamics Simulations

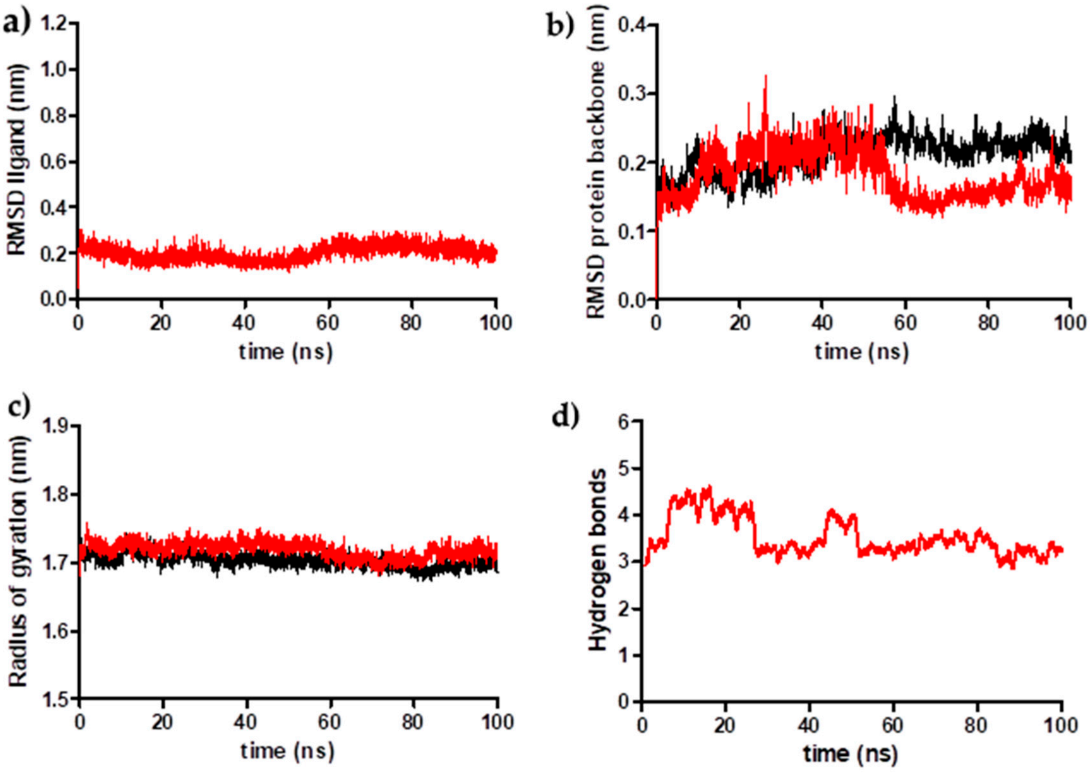

Figure S1. Analysis of the evolution of the complex of **1a** with 7PTF: **a)** RMSD of heavy atoms of the ligand; **b)** RMSD of protein backbone apo (black) and in complex with the ligand (red); **c)** radius of gyration of protein backbone apo (black) and in complex with the ligand (red); **d)** hydrogen bonds between the ligand and protein (1 ns moving average).

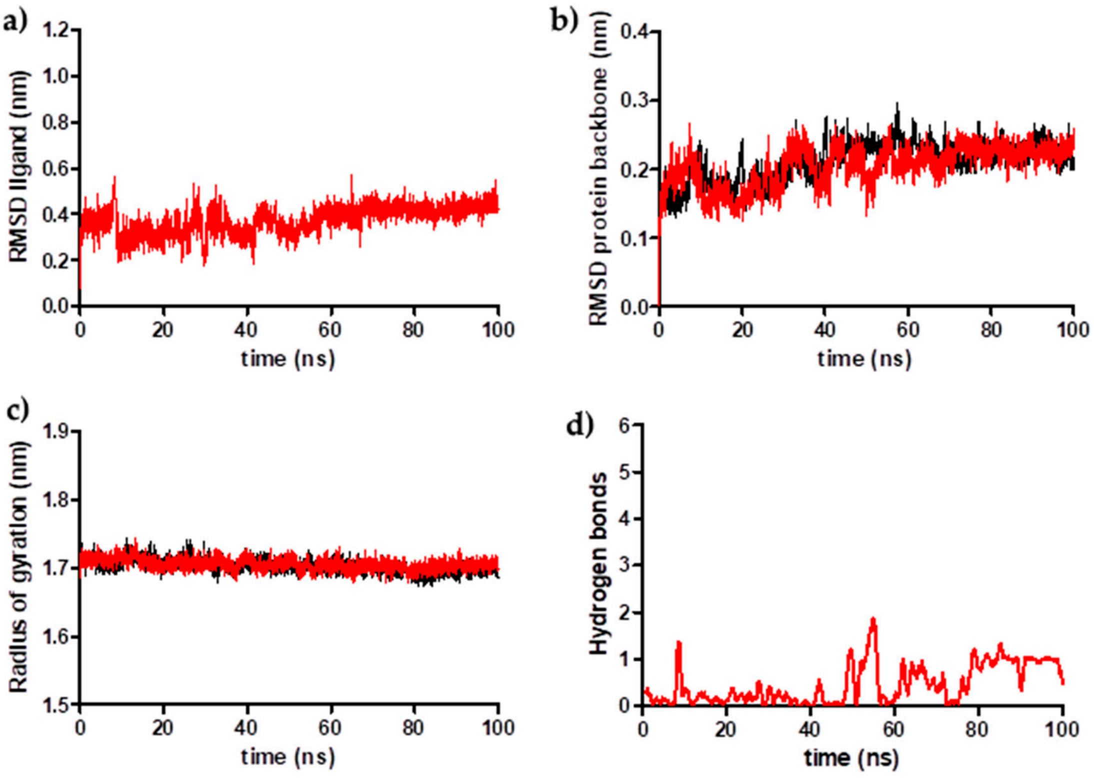

Figure S2. Analysis of the evolution of the complex of **1b** with 7PTF: **a)** RMSD of heavy atoms of the ligand; **b)** RMSD of protein backbone apo (black) and in complex with the ligand (red); **c)** radius of gyration of protein backbone apo (black) and in complex with the ligand (red); **d)** hydrogen bonds between the ligand and protein (1 ns moving average).

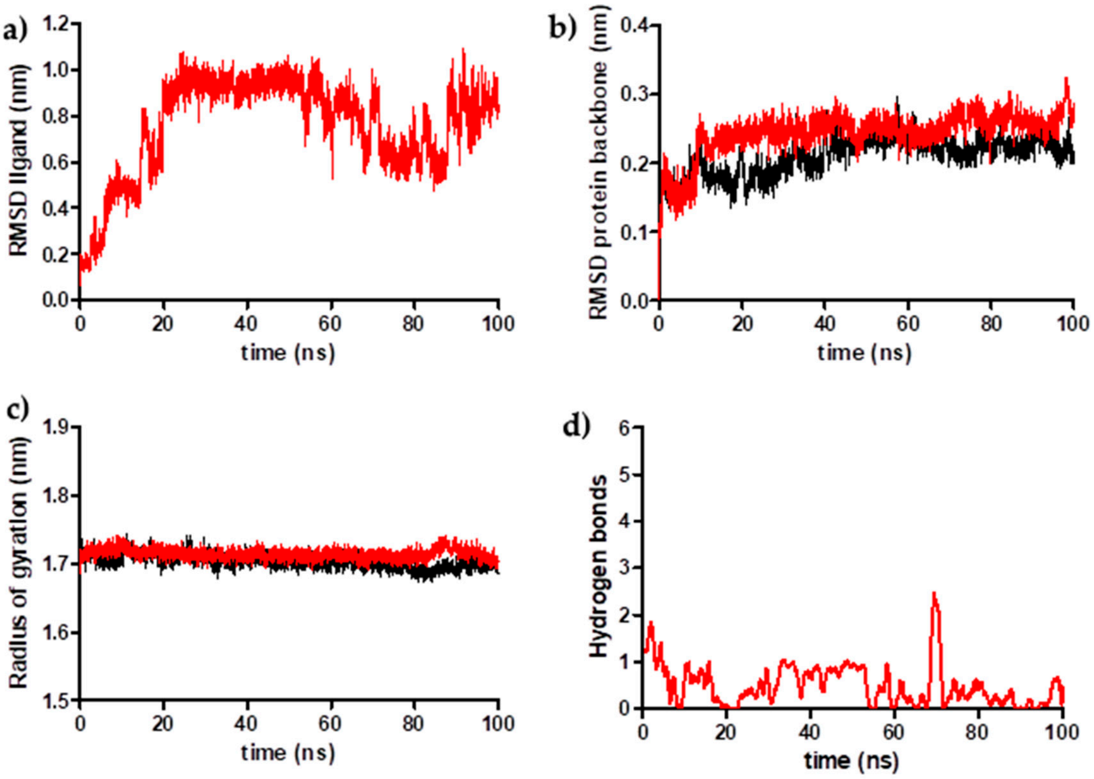

Figure S3. Analysis of the evolution of the complex of **1c** with 7PTF: **a)** RMSD of heavy atoms of the ligand; **b)** RMSD of protein backbone apo (black) and in complex with the ligand (red); **c)** radius of

gyration of protein backbone apo (black) and in complex with the ligand (red); **d)** hydrogen bonds between the ligand and protein (1 ns moving average).

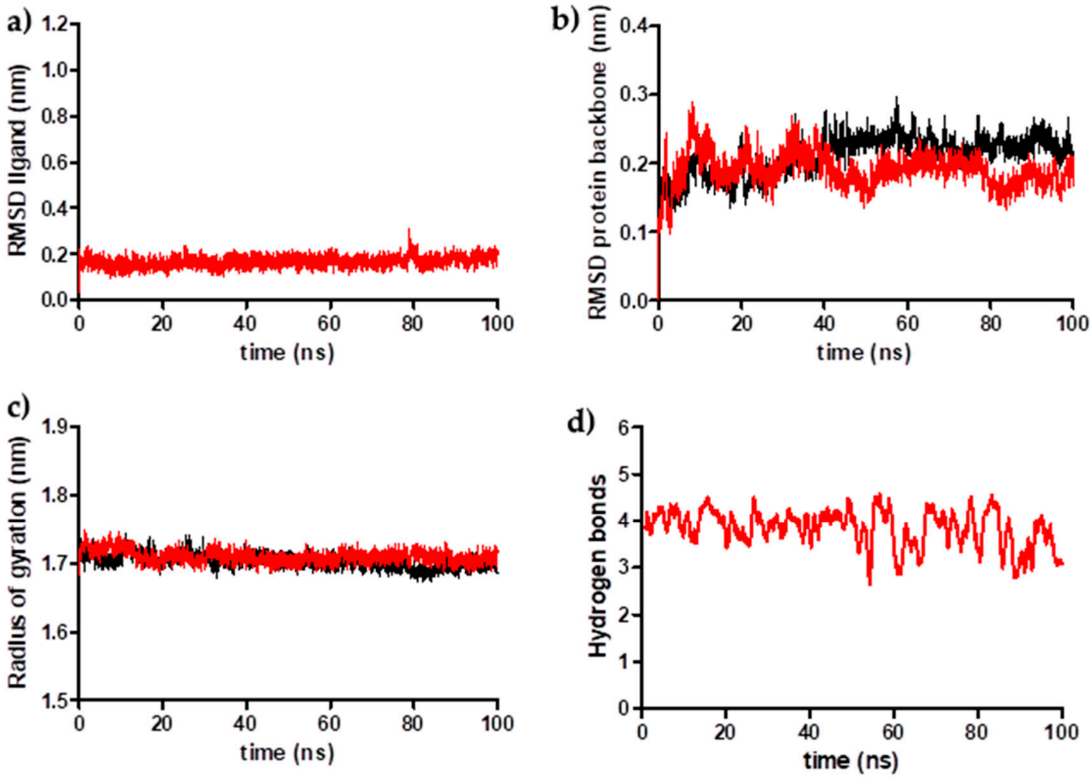

Figure S4. Analysis of the evolution of the complex of **1d** with 7PTF: **a)** RMSD of heavy atoms of the ligand; **b)** RMSD of protein backbone apo (black) and in complex with the ligand (red); **c)** radius of gyration of protein backbone apo (black) and in complex with the ligand (red); **d)** hydrogen bonds between the ligand and protein (1 ns moving average).

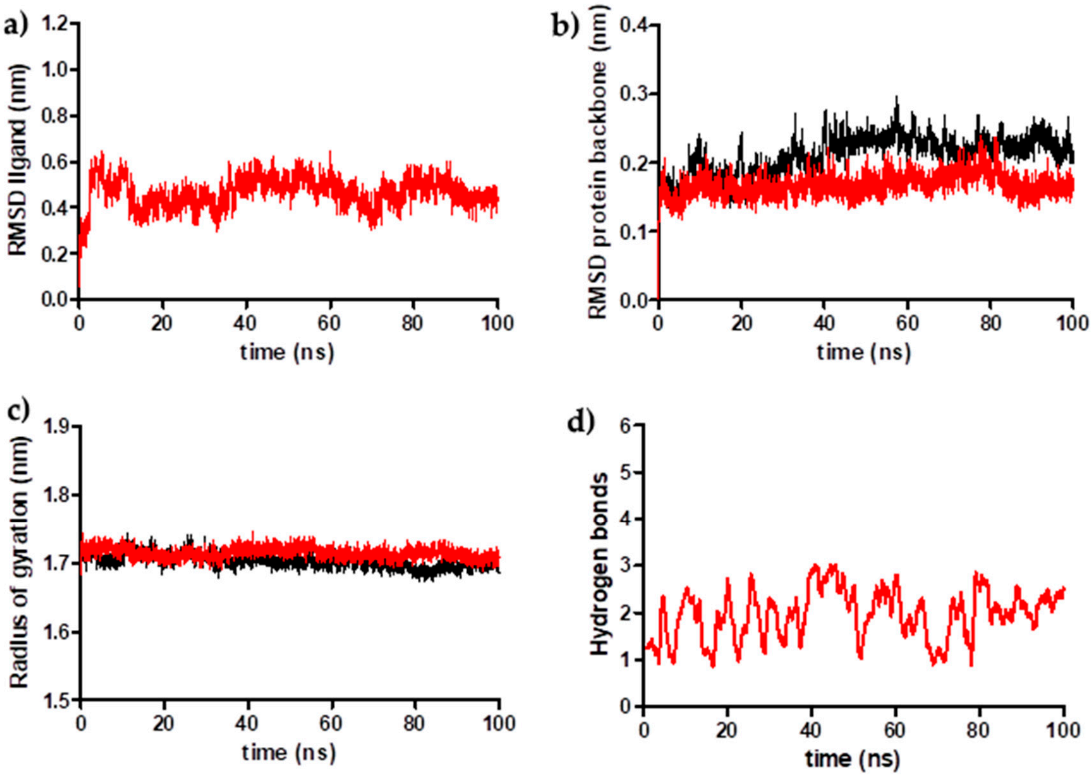

Figure S5. Analysis of the evolution of the complex of **1e** with 7PTF: **a)** RMSD of heavy atoms of the ligand; **b)** RMSD of protein backbone apo (black) and in complex with the ligand (red); **c)** radius of gyration of protein backbone apo (black) and in complex with the ligand (red); **d)** hydrogen bonds between the ligand and protein (1 ns moving average).

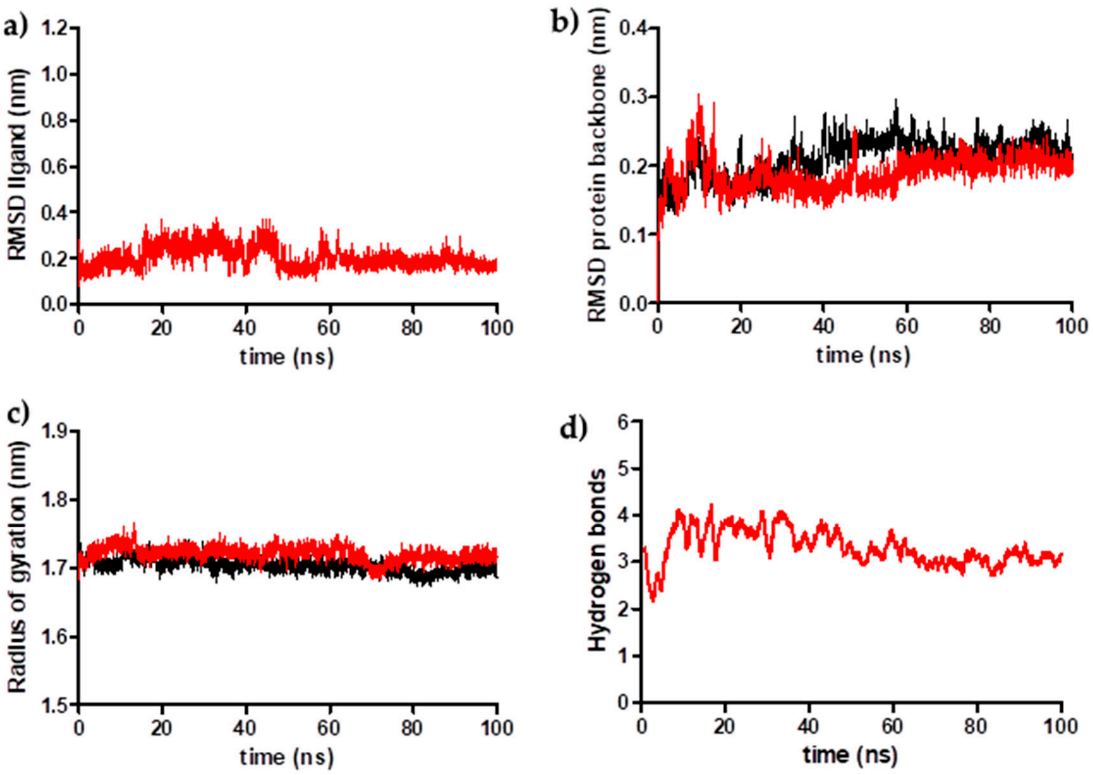

Figure S6. Analysis of the evolution of the complex of **1f** with 7PTF: **a)** RMSD of heavy atoms of the ligand; **b)** RMSD of protein backbone apo (black) and in complex with the ligand (red); **c)** radius of gyration of protein backbone apo (black) and in complex with the ligand (red); **d)** hydrogen bonds between the ligand and protein (1 ns moving average).

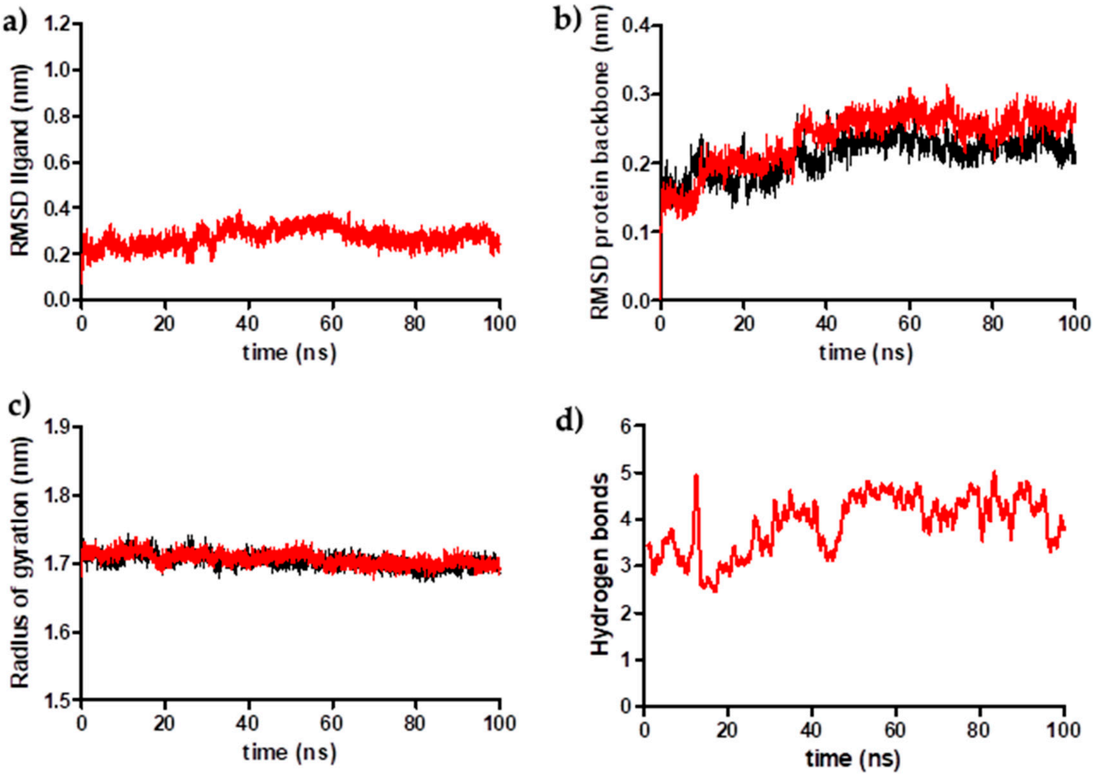

Figure S7. Analysis of the evolution of the complex of **1g** with 7PTF: **a)** RMSD of heavy atoms of the ligand; **b)** RMSD of protein backbone apo (black) and in complex with the ligand (red); **c)** radius of gyration of protein backbone apo (black) and in complex with the ligand (red); **d)** hydrogen bonds between the ligand and protein (1 ns moving average).

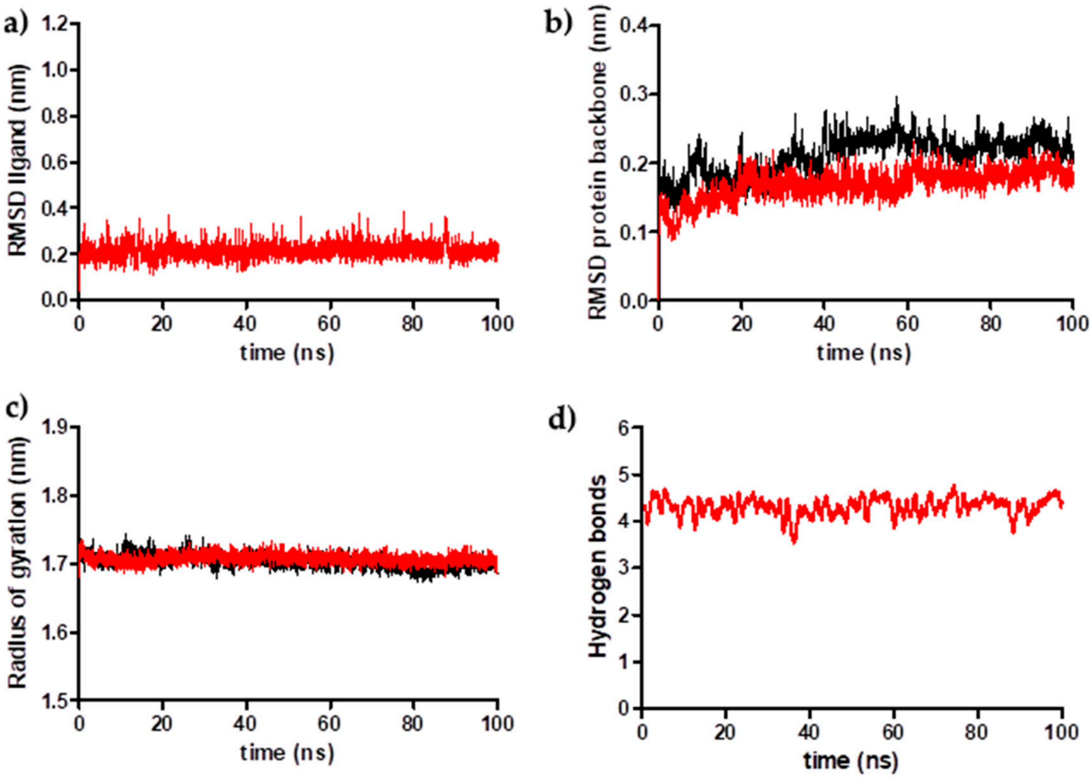

Figure S8. Analysis of the evolution of the complex of **novobiocin** with 7PTF: **a)** RMSD of heavy atoms of the ligand; **b)** RMSD of protein backbone apo (black) and in complex with the ligand (red);

c) radius of gyration of protein backbone apo (black) and in complex with the ligand (red); **d)** hydrogen bonds between the ligand and protein (1 ns moving average).
